# Evaluation of standard field and laboratory methods to compare protection times of the topical repellents PMD and DEET

**DOI:** 10.1038/s41598-018-30998-2

**Published:** 2018-08-22

**Authors:** Barbara Colucci, Pie Müller

**Affiliations:** 1 Epidemiology and Public Health Department, Swiss Tropical and Public Health Institute, Socinstrasse 57, PO Box, CH-4002 Basel, Switzerland; 2 University of Basel, Petersplatz 1, CH-2003 Basel, Switzerland

## Abstract

Mosquitoes are important vectors of pathogens, and travellers to disease endemic countries are advised to avoid bites by applying topical repellents. Topical repellents are typically tested either in the arm-in-cage (AIC) test under laboratory conditions or in the field, but not often under both conditions. We, therefore, investigated how two topical repellents, 15% *para*-menthane-3,8-diol (PMD) and 15% *N*,*N*-diethyl-3-methylbenzamide (DEET) compare against each other both in the AIC test against three species recommended by the World Health Organization (i.e. *Aedes aegypti*, *Anopheles stephensi* and *Culex quinquefasciatus*) and at two field sites in Switzerland, while using the same study participants in all experiments. In the field, the median complete protection time (CPT) was at least 6 hours for both PMD and DEET, while in the AIC test DEET slightly outperformed PMD. CPTs for DEET in the AIC test were 0.5, 2 and 2 hours against *Ae. aegypti*, *An. stephensi* and *Cx. quinquefasciatus*, respectively, and the corresponding median CPTs for PMD were 0.5, 1 and 0.5 hours. In conclusion, DEET slightly outperformed PMD in the AIC test, while the observed landing rates suggest the AIC test to underestimate efficacy of topical repellents in areas with lower landing pressure.

## Introduction

Biting mosquitoes (Diptera, Culicidae) are important vectors of several diseases such as malaria, filariasis and arboviral infections, including dengue, West-Nile, chikungunya and Zika, mainly in the tropical and subtropical regions but also increasingly in Europe as recent autochthonous cases of dengue and chikungunya have shown^1–4^. For most of the mosquito-borne diseases neither vaccines nor specific treatments exist and, therefore, travellers to disease endemic countries are advised to avoid mosquito bites, primarily by wearing appropriate clothing and by applying topical repellents on the bare skin. Topical repellents may provide good protection against mosquito bites over several hours depending on the active ingredient and its concentration^5–9^.

The most widely used active ingredient in commercially available mosquito repellents is the synthetic compound *N*,*N*-diethyl-3-methylbenzamide (DEET) which is generally regarded as the “gold standard” due to its high efficacy against a broad range of insects^10,11^. Although DEET is deemed nontoxic if used correctly, concerns have been raised about its safety^12^. Moreover, DEET has plasticising properties, a strong smell and may even cause discomfort, particularly when applied at higher dosages. Users may, therefore, wish to buy repellents containing alternative actives. An alternative compound which has been shown to be effective against a range of mosquito species is *para*-menthane 3,8-diol (PMD)^13,14^. PMD originates from the residuum of the hydrodistillation of the leaves of the lemon eucalyptus tree *(Corymbia citriodora)* but may also be produced synthetically.

Several studies have compared the efficacy of PMD against DEET, yet the existing data are somewhat difficult to read because the formulations tested were either commercially available products, containing additional compounds that potentially also influence the efficacy of the repellent^15^, or the concentrations of PMD and DEET of the formulations applied within the same study differed^16,17^, or both^14,15,18,19^. Some studies had also very low sample size, including only 3 or even fewer study participants^15,16,18^, casting doubts on the generalisation of these results to a wider population.

The efficacy of topical mosquito repellents is usually tested following various national or international guidelines (e.g.^20,21^). Under laboratory conditions, the efficacy of mosquito repellents is typically evaluated in the so-called “arm-in-cage” (AIC) test. In the AIC test, according to the guidelines, a forearm of a study participant is treated with a defined amount of the repellent formulation (e.g. 1 ml per 600 cm^2^). Then the participant exposes the treated forearm at regular intervals (e.g. every half hour) to a number (e.g. 200) of host-seeking female mosquitoes for a defined exposure period (e.g. 3 minutes) in a cubic cage (e.g. 64,000 cm^3^). The endpoint is usually the complete protection time (CPT) or the relative protection *(%p)*. CPT corresponds to the time from the application of the formulation until its failure, while the relative protection is the percentage protection provided as compared to an untreated forearm. In the field, similar endpoints may be determined on the basis of mosquitoes landing or biting on an exposed skin area, usually the lower leg, while the mosquitoes are collected by aspiration allowing for the identification of the mosquito species in a laboratory.

While the guidelines recommend conducting efficacy studies both under laboratory and field conditions, mosquito repellents are hardly being compared side-by-side in both settings. For example, searching the literature database PubMed for insect repellents using the Medical Subject Headings terms “insect repellents”, “Culicidae” and “terpin”, which includes PMD, together with the published studies mentioned in the review of Carroll & Loye^14^ yields 9 studies done either in the laboratory (N = 4)^16,22–24^ or in the field (N = 5)^13,16,17,19,25^ but only 1 study that evaluated the repellent’s efficacy under both laboratory and field conditions^14^. This raises the question as to how protection efficacy measured under laboratory conditions compares to the efficacy under real conditions of use. A better understanding of this relationship would allow for validating and optimising existing laboratory assays, particularly since field studies are time and cost intensive.

In order to shed more light on the relationship between the protection provided by a topical repellent against mosquito bites in laboratory and field settings, and to evaluate the efficacy of the active ingredient PMD against DEET under equal conditions, we conducted an experimental study that compares the protection times of 15% ethanolic solutions of PMD and DEET by exposing the same 18 study participants using similar parametres both in the field and in the laboratory.

## Methods

### Study design

This is a comparative study of laboratory and field methods to determine the efficacy of topical mosquito repellents. Two solutions of 15% DEET and 15% PMD were tested on human subjects as it utilised the repellent end-user in the testing process. The same volunteers tested the same formulations both under laboratory and field conditions. In order to generate comparable data the parameters in the field and in the laboratory experiments were kept as identical as possible, while following World Health Organization (WHO)^20^ and the United States Environmental Protection Agency (US EPA)^21^ guidelines. The primary outcome was the complete protection time (CPT) and relative protection (*%p*) as compared to a negative control over 6 hours post application of the repellent.

### Study participants

Eighteen study participants, 8 women and 10 men, showing low or no skin reactions against mosquito bites were recruited at the University of Basel and selected upon a face-to-face interview. The age of the study participants ranged between 21 and 37 years. The study was approved by the Ethics Commission of Northwest and Central Switzerland (Study no: EKNZ 2015-238) and all study participants signed the informed consent form. To avoid unwanted bias the participants were asked to avoid alcohol and products such as perfume, eau de cologne and lotions for at least 12 hours before and during the experiments. During the experiments the participants were also asked to avoid rubbing, touching or wetting the repellent-treated area as well as any activity that might lead to increased perspiration.

### Test formulations

The test formulations consisted of ethanolic solutions of 15% DEET (m/m) and 15% PMD (m/m) alongside a negative control of 70% ethanol (v/v). The test formulations were kindly provided by Vifor Consumer Health SA (Villars-sur-Glâne, Switzerland). DEET was chosen because it represents the gold standard and still remains the most effective mosquito repellent^11,26^. PMD was chosen as this active ingredient has received less attention in the literature, is a natural based repellent and has been reported to be similarly effective as DEET^18,19,27^.

### Field experiments using the human landing catch method

In the field, the repellents were evaluated at two locations in Switzerland that differ in their ecology; the Langholz forest (E 7.87170, N 47.28607), a restored forest area in the Canton of Aargau and in the meadows of the Thurauen Nature Reserve (E 8.59496, N 47.59600) between the rivers Thur and Rhine in Ellikon am Rhein, Canton of Zürich. Both areas usually display a relatively high abundance of mosquitoes, while the Langholz forest also shows a very high mosquito species diversity^28^. The Thurauen are particularly known for the presence of the flood water mosquito *Aedes vexans*^29,30^. During the study, both areas were neither treated with insecticides nor were they subject to any other vector control measure.

The efficacy of the repellent formulations were first assessed in the Langholz forest between 20 July and 12 August 2015 and then in the Thurauen Nature Reserve between 17 and 30 August 2015. Using the human landing catch (HLC) method, observations were made hourly for 30 minutes over 6 hours, starting 1 hour post application of the formulations at 17:00 hours and 14:15 hours in Langholz and Thurauen, respectively.

In the experiments, the 18 study participants were split into 3 groups of 6 (Supplementary Fig. S1). Each group tested the 2 formulations, 15% PMD and 15% DEET, and the negative control containing 70% ethanol. Over 3 consecutive days each person received each treatment once and 2 participants tested the same treatment on a given day. The sequence of treatment allocation was organised in a Williams balanced Latin Square design^31^ to minimise any bias due to first-order carryover effects, while on the first day each study participant was randomly assigned to one of the 6 possible schedules (Supplementary Fig. S1) by means of drawing lots. In addition, to avoid carryover effects of residual repellents the other leg was treated on the subsequent day.

In preparation of the field experiments, the test surface (i.e. the bare lower leg) of the study participants were washed with neutral soap, rinsed, dried and swabbed with Arixtra wipes containing 70% isopropanol (Sanofi-Synthelabo, Meyrin, Switzerland). One of the lower legs was then treated with either one of the two repellent formulations or the negative control at a rate of 1 ml per 600 cm^2^. The application volume was estimated on the basis of the surface area, calculated as the average of the circumferences just below the knee, the calf and the ankle, multiplied by the length of the lower leg, measured from below the knee to the ankle.

**Figure 1:**
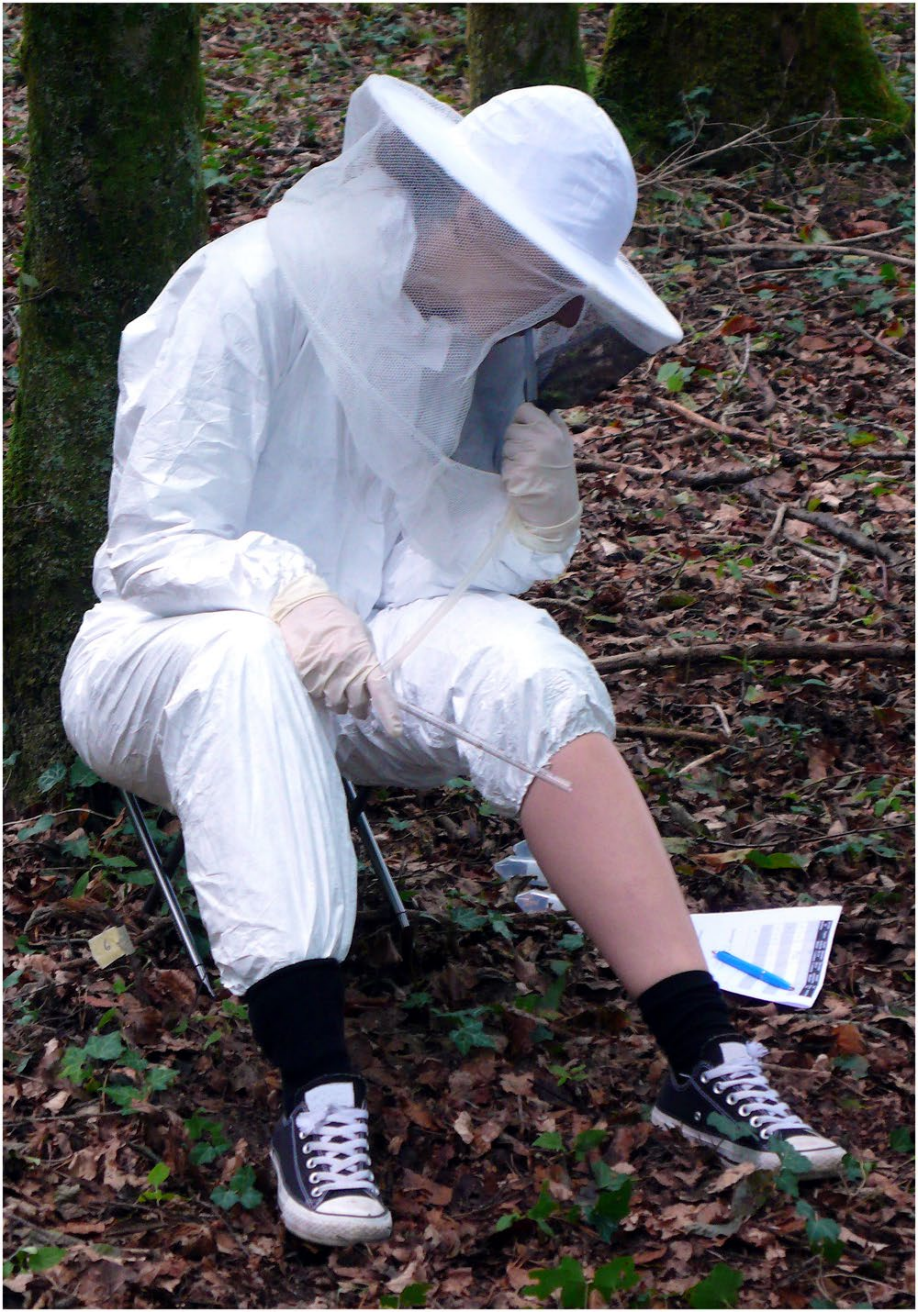
The human landing catch (HLC) method for measuring the efficacy of topical repellents under field conditions. Mosquitoes alighting on the treated and exposed lower leg were aspirated by a mouth aspirator and kept in Falcon tubes for species identification in the laboratory. Apart from the treated area the whole body was fully protected by a jump suit, a bee keeper’s hat and latex gloves through which mosquitoes cannot bite.

Sixty minutes after application of the treatment, the study participants were assigned to one of 6 positions that were set at least 20 m apart, as recommended in the WHO guidelines^20^, in order to avoid bias due to competition in attractiveness to the mosquitoes. With the exception of the treated lower leg the whole body was fully protected from mosquito bites by a white jump suit, a bee keeper’s hat and latex gloves through which mosquitoes could not bite. During an exposure period of 30 minutes the study participants sat on a stool and collected any mosquito alighting on the exposed lower leg using a mouth aspirator (Fig. 1). Collected insects were transferred to 50 ml Falcon tubes (Corning Life Sciences, Amsterdam, Netherlands) covered with mesh. When natural light conditions were insufficient to carry out collections the study participants used a head torch (7 LED, Cyba Headlight, Edelrid, Isny, Germany). Before the field experiments, the study participants were trained in collecting mosquitoes with a mouth aspirator in an experimental room containing free-flying mosquitoes.

An acoustic signal from a horn indicated the participants the start and the end of the 30 minutes exposure period. Following the exposure the study participants retreated from their position for 30 minutes before they moved to the next position, repeating the process above until they had 6 exposures over 6 hours. As with the treatment allocation, the sequence of rotation between the positions followed a Williams balanced Latin Square design and was randomly assigned to the study participants at the beginning of each session by drawing lots.

In addition to the mosquito collections by the study participants, mosquito traps were set in the study area to measure overall mosquito presence. The traps ran during the same 6 hours the study participants tested the repellents, yet the traps were set at least 20 metres away in order to avoid unwanted attraction of mosquitoes away from, or towards to, the study participants.

In the Langholz forest trapping was done with one BG-Sentinel trap baited with BG lure (Biogents AG, Regensburg, Germany) and one Centers for Disease Control (CDC) Miniature Light Trap (Model 512, John W. Hock Company, Gainesville, FL, USA) equipped with dry ice but with the light bulb removed. In the Thurauen Nature Reserve there was no access to dry ice and, therefore, the CDC light trap was replaced by a second BG-Sentinel trap baited with CO_2_ from a bottle.

All collected mosquitoes were identified at the Swiss Tropical and Public Health Institute (Swiss TPH) with the aid of a stereo microscope using the identification keys of Schaffner *et al*.^32^ and Becker *et al*.^33^. If it was not possible to identify a mosquito specimen on the basis of morphological characteristics, for example when it was damaged or a member of a species complex, the specimen was processed and sent to Mabritec AG (Riehen, Switzerland) for molecular identification. The molecular identification method used is based on matrix assisted laser desorption/ionization time-of-flight mass spectrometry (MALDI-TOF MS), an emerging methodology for the identification of arthropods^34^.

During the experiments, additional physical parameters were recorded, including wind speed, temperature and weather conditions. Experiments were only carried out as long as the weather was dry. Average wind speeds recorded in both field trials were 0.25 m/s, ranging between 0 and 2.7 m/s, while the mean temperature was 24.8 °C, ranging from 17.9 °C to 33.5 °C.

### Laboratory experiments using the arm-in-cage test

**Figure 2:**
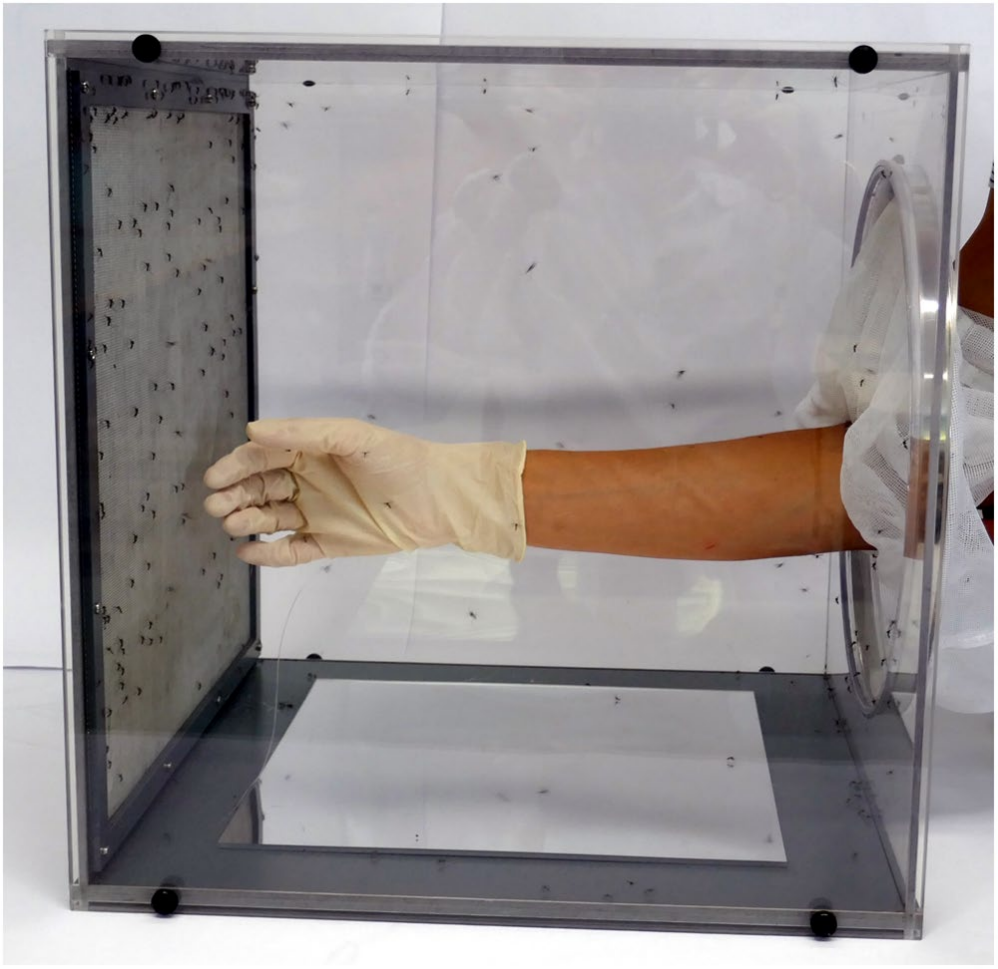
The arm-in-cage (AIC) test for measuring the efficacy of topical mosquito repellents under laboratory conditions. Hungry female mosquitoes are contained in a test cage and the repellent is applied to the forearm between the wrist and elbow, while the hand is protected by a latex glove through which the mosquitoes cannot bite.

The laboratory experiments were conducted at Swiss TPH in Basel between 5 August and 21 November 2015, following the WHO guidelines for the arm-in-cage (AIC) test^20^. In the AIC test, the protection time of a repellent is assessed by exposing a treated forearm to hungry mosquitoes at regular intervals. The cages measured 40 cm × 40 cm × 40 cm and were made of clear acrylic glass sides with an opening on the front side. At the bottom of the cage was a mirror positioned allowing for observation of mosquitoes landing on the lower side of the arm. The back side of the cage was made of a fine metal grid to ensure air supply during the experiments (Fig. 2).

The test cages contained 200 host seeking 5 to 10-day-old females of one of the 3 mosquito species; *Ae. aegypti, An. stephensi* or *Cx. quinquefasciatus*. The three mosquito species are WHO recommended model organisms^20^. Adult mosquitoes were fed with 10% sucrose solution and water *ad libitum*. Testing and rearing conditions for all mosquito colonies were 26.8 ± 1.2 °C (mean ± SD) and 64.8% ± 7.2% relative humidity (mean ± SD) and a 12:12 (light:dark) photoperiod. Male and female mosquitoes were kept in the same rearing cages to allow mating to occur.

Experiments with *Ae. aegypti* were performed under artificial light at an intensity of 639 Lux, while tests with *An. stephensi* and *Cx. quinquefasciatus* were conducted under subdued light at an intensity of 49 Lux to mimic the conditions according to the mosquito species diurnal biting patterns. Twelve hours before the experiment, the sugar water was removed from the cage and the mosquitoes had only access to water.

Before exposure to the mosquitoes the forearm was washed with odourless soap, dried with a towel, swabbed with an Arixtra isopropanol wipe and then dried again. Then, to assess the readiness of the mosquitoes to land, the forearm of a study participant was exposed in the experimental cage for 60 seconds or until 10 landings were counted. A landing was defined as a mosquito alighting on the skin and remaining for at least 2 seconds. After measuring the landing activity with the untreated forearm the forearm was treated from wrist to elbow with either 15% PMD or 15% DEET at an application rate of 1 ml per 600 cm^2^. In order to estimate the application volume the surface area of the forearm was calculated as the average circumference of the elbow, wrist and middle of the forearm multiplied by the distance between the wrist and the cubital joint. Each volunteer tested only one repellent per day. Thirty minutes after application of the repellent the participant exposed the treated forearm in the test cage for 3 minutes or until 10 mosquitoes landed. The procedure was then repeated every 30 minutes over 6 hours. The duration until the first, second and tenth landing of a mosquito on the treated forearm was noted. During the exposure time volunteers were allowed to shake off the mosquitoes before they started biting, preventing an excessive number of bites. At the end of the experiment the arm was again washed and dried as before, and a second control measurement of the mosquitoes’ landing activity was taken.

### Data analysis

Raw data recorded on paper forms were entered into a Microsoft Excel 2010 spread sheet. Each entry was double-checked by two different persons and the records were inspected for outliers and inconsistencies. Statistical analysis was performed in the open source package R version 3.4.2^35^ and graphs were produced with the R package “ggplot2”^36^.

The endpoint measured in the experiments was the number of mosquitoes landing on the bare skin during each exposure period. Based on the number of landings and exposure times two outcome measures were estimated following the WHO guidelines^20^: the complete protection time (CPT) and the percentage protection *(%p)* over time.

Here, CPT is defined as the time elapsed between the application of the repellent and the first mosquito landing. Average CPTs (median and 95% confidence interval; 95% CI) over all study participants were estimated based on a Kaplan-Meier survival analysis implemented in the R package “survival”^37,38^ and compared between the two repellent formulations using the Mantel-Haenszel test, including a stratification term for study participants.

*%p* was calculated as the reduction in landings by the treatment when compared to the negative control over all exposures using equation (1).

1 $$ \% p=\frac{C-T}{C}\times 100$$

where *T* is the average number of mosquitoes landing on the surface per second in a test and *C* is the average number of mosquitoes per second landing on the skin surface treated with the negative control in the field experiment or the untreated forearm in the AIC test.

Landing rates were estimated on the basis of generalised linear models (GLM) with a negative binomial distribution and a log link function. Separate GLMs were modelled for the field and laboratory experiments. For the field data, landing rates were modelled as a function of treatment, time post application and location. In addition, interaction terms were introduced into the model to account for non-additive effects between treatment, time and location. Similarly to the field experiments, landing rates in the AIC test were modelled as a function of treatment, time post application and mosquito strain, and interaction terms were introduced to account for non-additive effects. In both models an offset term with the log of the exposure time was introduced to capture the differences in exposure times between tests. For example, the study participants were allowed to conclude an exposure after 10 landings in the AIC test, leading to different exposure times.

As for the landing rates average *%p* over the 6 hours test period was estimated using GLMs to model the total number of landings per person per second and then compared between 15% PMD and 15% DEET. The same approach was also used to compare the control landing rates in the AIC test before and after the experiments.

For statistical testing the level of significance was set at *α* = 0.05.

## Results

From the 18 study participants initially enrolled, one female dropped out during the study; hence only 17 participants were included in the data analysis. In addition, for the field experiments in the Thurauen Nature Reserve, one measurement is missing for 15% PMD and one for the negative control due to two study participants being on sick leave.

### Mosquito species composition in the field

In total, 227 landings were recorded in the field experiments using HLC with an approximately equal number of mosquitoes counted in the Langholz forest (*n* = 115) and the Thurauen Nature Reserve (*n* = 112). From the 227 recorded landings, 118 mosquitoes were actually collected in tubes and subjected to a taxonomical identification (Table 1). Only 1 out of the 118 mosquitoes could not be identified, neither to species nor genus level. In addition to the HLC, mosquitoes were also trapped by BG-Sentinel traps and a CDC miniature light trap (Table 1) that ran in parallel during the experiments. In the BG-Sentinel traps 59 and 8 specimens were collected in the Thurauen Nature Reserve and the Langholz forest, respectively, while the CDC light trap in the Langholz forest caught 272 specimens. As with the HLC method, most of the specimens from the traps could be identified to either species or at least to genus level (i.e. 98.2%, *n* = 339).

**Table 1:** Species composition of mosquitoes collected over the course of the field experiments by HLC, BG-Sentinel trap and the CDC miniature light trap.

Species	HLC			BG-Sentinel trap			CDC light trap
	Langholz	Thurauen	Total	Langholz	Thurauen	Total	Langholz
*Aedes annulipes*	0	1 (1.8)	1 (0.8)	0	0	0	0
*Aedes cinereus/geminus* ^a^	32 (52.5)	13 (22.8)	45 (38.1)	0	21 (35.6)	21 (31.3)	14 (1.1)
*Aedes vexans*	4 (6.6)	37 (64.9)	41 (34.7)	0	21 (35.6)	21 (31.3)	0
*Aedes sticticus*	7 (11.5)	0	7 (5.9)	2 (25.0)	0	2 (3.0)	3 (1.1)
*Anopheles claviger*	0	0	0	0	1 (1.7)	1 (1.5)	0
*Anopheles maculipennis*	1 (1.6)	0	1 (0.8)	0	0	0	115 (42.3)
*Anopheles plumbeus*	9 (14.8)	3 (5.3)	12 (10.2)	1 (12.5)	0	1 (1.5)	27 (9.9)
*Anopheles* sp.	0	0	0	0	0	0	1 (0.4)
*Coquillettidia richiardii*	6 (9.8)	1 (1.8)	7 (5.9)	0	1 (1.7)	1 (1.5)	19 (7.0)
*Culex martinii*	2 (3.3)	0	2 (1.7)	0	3 (5.1)	3 (4.5)	2 (0.7)
*Culex pipiens*	0	2 (3.5)	2 (1.7)	5 (62.5)	11 (18.6)	16 (23.9)	82 (30.1)
*Culiseta galphyroptera*	0	0	0	0	0	0	4 (1.5)
Not identified	0	0	0	0	1 (1.7)	1 (1.5)	5 (1.8)
Total	61	57	118	8	59	67	272

The figures show the number of specimens collected and percentage composition (%). ^a^The sibling species could not be distinguished and were counted as one single taxon.

The predominant taxa were *Ae. cinereus/geminus*, a species complex, and *Ae. vexans*, followed by *An. plumbeus*. *Ae. cinereus/geminus* was the most common taxon in the Langholz forest and *Ae. vexans* was the most common species in the Thurauen Nature Reserve. Together, the two taxa make 70.5% of the overall species identified in the HLC. In terms of biting activity, *Ae. vexans* was predominantly active in the late afternoon, while *Ae. cinereus/geminus* was mainly active around and after sun set. In contrast, the identified *An. plumbeus* specimens were mainly active during the crepuscular period.

### Human landing rates

**Figure 3:**
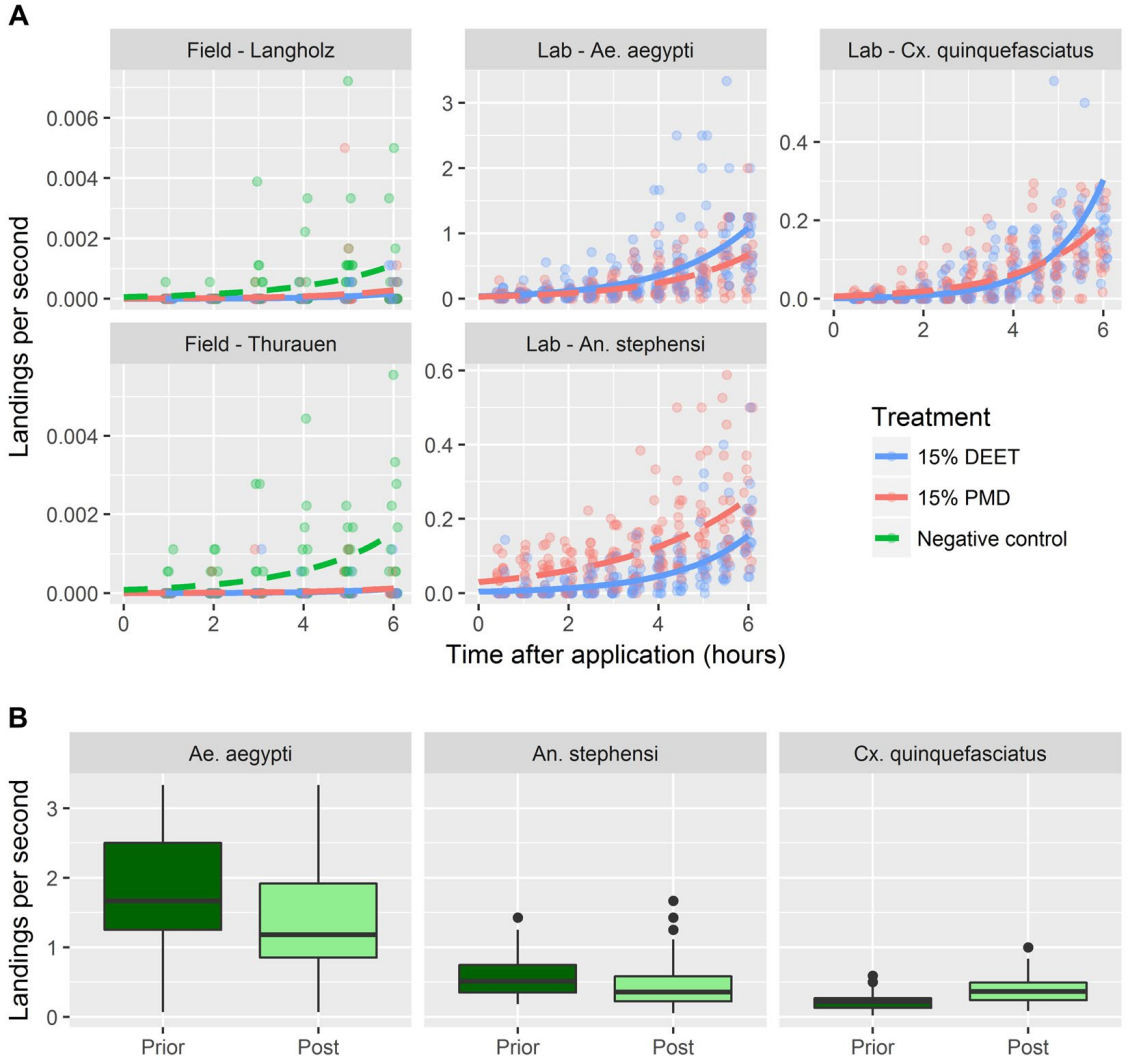
Landing rates in the laboratory and field experiments. (**A**) Landing rates as a function of time. While in the field experiments the participants tested a negative control over 6 hours, the negative control in the laboratory AIC test was done once before and once after the actual experiment with the treated arm; hence, unlike in the field experiments, no curves are plotted for the negative controls in the AIC test. For ease of visibility the points are jittered along the x-axis. The individual model parameters of the underlying GLMs are given in Tables 2 and 3. (**B**) Landing rates measured in the AIC test on the untreated forearms prior and post treatment with the repellent. The boxes represent the interquartile distances (IQD), while the centre lines through each box show the medians. The dots indicate outliers and the whiskers extend to the extreme values of the data, calculated as ± 1.5 × IQD from the median. For ease of readability a single outlier showing a landing rate of 10 landings per second in *Ae. aegypti* in the first control has been removed from the graphic.

Human landing rates differed considerably between the laboratory and the field experiments, both on the treated and untreated surfaces. In the negative control, the average number of landings per second (mean and range) was 0.00046 (0.0–0.0072) in the Langholz forest and 0.00057 (0.0–0.0056) in the Thurauen Nature Reserve (Fig. 3A). In contrast, in the AIC tests the average number of landings per second were several magnitudes higher; 1.68 (0.07–10.0), 0.54 (0.05–1.67) and 0.31 (0.2–1.0) landings per second for *Ae. aegypti*, *An. stephensi* and *Cx. quinquefasciatus*, respectively (Fig. 3B). The landing rates in the negative controls in the field experiments showed an increase towards the end of the 6 hours test period, suggesting mosquitoes becoming more active towards the end of the day (Fig. 3A). In the AIC the negative controls performed before and after the actual experiment also showed differences in landing activities. In *Ae. aegypti* (rate ratio between prior and post experiment; RR_prior-post_ = 1.44, *p* < 0.001) and *An. stephensi* (RR_prior-post_ = 1.36; *p* < 0.01) the mosquitoes were less active at the end of the test, while in *Cx. quinquefasciatus* (RR_prior-post_ = 0.61; *p* < 0.001) the mosquitoes showed the opposite behaviour (Fig. 3B).

In the field, the landing rates for all conditions increased over time but were lower in the 15% PMD and 15% DEET arms, while statistically there was no significant difference between the landing rates of PMD and DEET (Fig. 3A and Table 2). Across the three different lab colonies tested in the AIC test, there was no consistent difference in the landing rates between PMD and DEET (Fig. 3A and Table 3): DEET outperformed PMD against *An. stephensi* (RR_PMD-DEET_ = 7.55; p < 0.001) and to a lesser extent also against *Cx. quinquefasciatus* (RR_PMD-DEET_ = 4.14; *p < *0.001) where PMD was catching up with DEET towards the end of the 6 hours exposure period. For *Ae. aegypti* the difference between PMD and DEET was statistically not significant (RR_PMD-DEET_ = 0.82; *p* = 0.358).

**Table 2:** Generalised linear model to estimate and predict the average mosquito landing rates in the field experiments.

Parameter	β (log_2_)	SE (β) (log_2_)	*Z*-value	*P*-value
(Intercept)	−9.378	0.416	−22.542	<0.001
Location (Langholz)	−0.310	0.299	−1.037	0.3
Treatment (15% DEET)	−4.035	1.306	−3.090	<0.01
Treatment (15% PMD)	−3.133	1.024	−3.061	<0.01
Time (post application)	0.483	0.092	5.254	<0.001
Interaction term: Location (Langholz) × treatment (15% DEET)	0.779	0.667	1.167	0.243
Interaction term: Location (Langholz) × treatment (15% PMD)	1.159	0.606	1.914	0.056
Interaction term: Treatment (15% DEET) × time	0.235	0.250	0.941	0.347
Interaction term: Treatment (15% PMD) × time	0.101	0.199	0.506	0.613

β: regression coefficient; SE(β): standard error of β. The intercept corresponds to the reference that is the number of landings per second in the negative control in the Thurauen Nature Reserve.

**Table 3:** Generalised linear model to estimate and predict the average mosquito landing rates in the arm-in-cage experiments.

Parameter	β (log_2_)	SE(β) (log_2_)	*Z*-value	*P*-value
(Intercept)	−3.239	0.150	−21.627	<0.001
Treatment (15% PMD)	−0.195	0.213	−0.919	0.358
Lab colony *(An. stephensi)*	−2.288	0.235	−9.735	<0.001
Lab colony *(Cx. quinquefasciatus)*	−3.253	0.253	−12.871	<0.001
Time (post application)	0.554	0.041	13.671	<0.001
Interaction term: Treatment (15% PMD) × lab colony *(An. stephensi)*	2.217	0.318	6.977	<0.001
Interaction term: Treatment (15% PMD) × lab colony *(Cx. quinquefasciatus)*	1.615	0.340	4.755	<0.001
Interaction term: Treatment (15% PMD) × time (post application)	−0.048	0.057	−0.832	0.405
Interaction term: Lab colony *(An. stephensi)* × time (post application)	0.056	0.061	0.914	0.361
Interaction term: Lab colony *(Cx. quinquefasciatus)* × time (post application)	0.329	0.064	5.121	<0.001
Interaction term: Treatment (15% PMD) × lab colony *(An. stephensi)* × time (post application)	−0.206	0.084	−2.441	0.015
Interaction term: Treatment (15% PMD) × lab colony *(Cx. quinquefasciatus)* × time (post application)	−0.261	0.088	−2.961	<0.01

β: regression coefficient; SE(β): standard error of β. The intercept corresponds to the reference that is the number of landings per second in the DEET treatment in *Ae. aegypti*.

### Complete protection time

**Figure 4:**
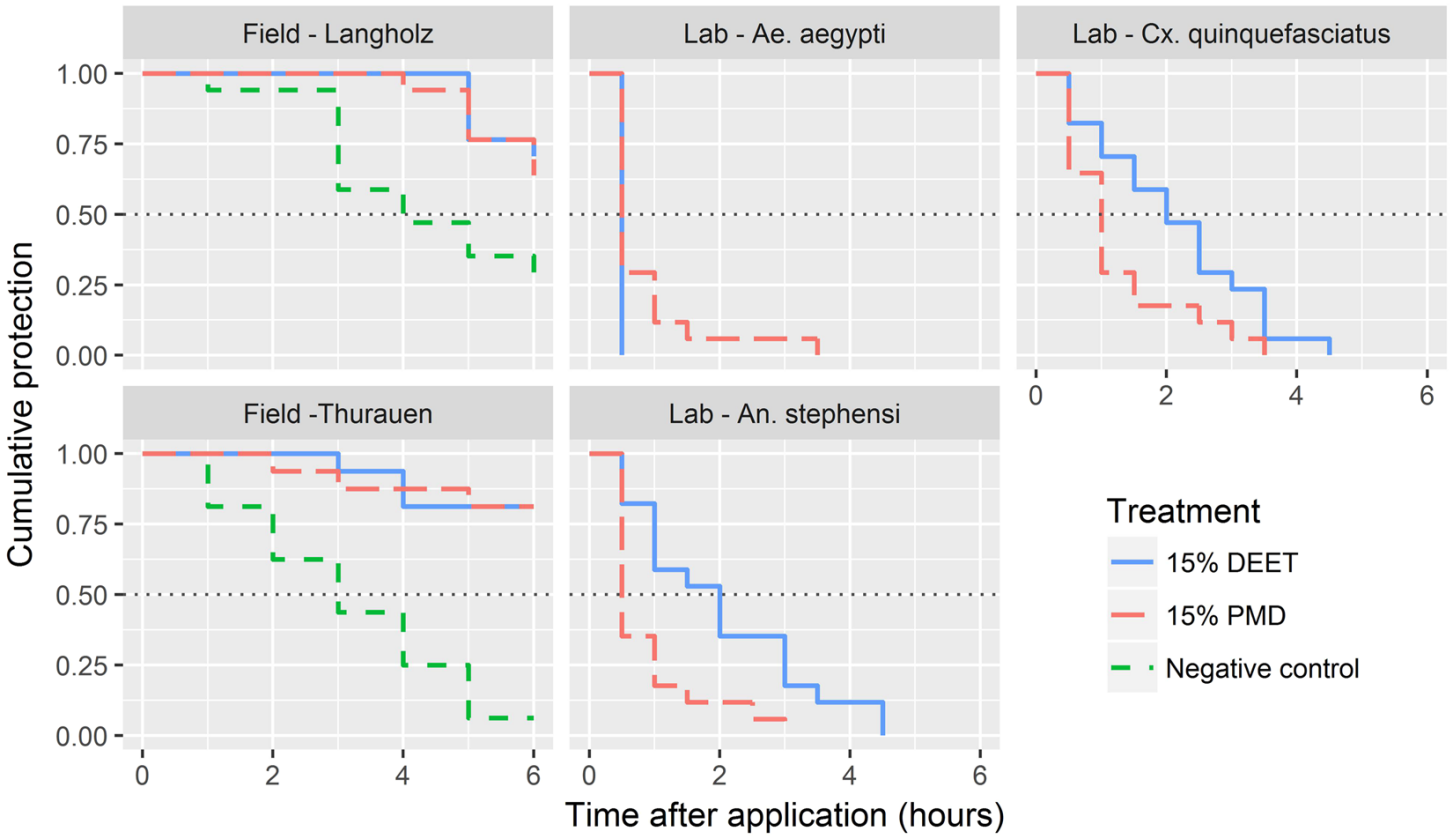
Cumulative complete protection time (CPT). The lines show the proportion of study participants remaining completely protected by the applied formulation as a function of time post application. While in the field experiments the participants tested a negative control over 6 hours, the negative control in the AIC test was only done before and after the experiment; hence no curves are plotted for the negative controls in the AIC test. The intersections between the survival curves and the dotted lines, representing 50% cumulative protection, indicate the median CPTs.

CPT corresponds to the time from the application of the formulation until failure of the repellent, measured here as the first mosquito landing on the treated area. CPTs were much higher in the field experiments and are in agreement with the low landing rates observed in both the Langholz forest and the Thurauen Nature Reserve. In fact, more than 50% of the participants had not received a single landing even at 6 hours post application of the repellent formulations (Fig. 4; Supplementary Fig. S2). Despite the low numbers of landings, even recorded in the negative control, the time to the first landing was much longer in the treatment arms as compared to the negative control arm (Control Langholz: median CPT = 4 hours, *χ*^2^ = 17.6, df = 2, *p* < 0.001; control Thurauen: median CPT = 3 hours, *χ*^2^ = 23.2, df = 2, *p* < 0.0001; Fig. 4).

The picture for the CPTs in the AIC test is quite different but reflects the observations made for the landing rates (Fig. 4). Here, CPTs for DEET were 30 minutes against *Ae. aegypti*, 2 hours against *An. stephensi* (95% CI: 1–3 hours) and 2 hours (95% CI: 1.5–3.5 hours) against *Cx. quinquefasciatus*. The median CPTs for PMD were lower than for DEET against *An. stephensi* and *Cx. quinquefasciatus* with 0.5 hour (95% CI: 0.5–1.0 hour; *χ*^2^ = 0.3, *p* < 0.05) and 1 hour (95% CI: 0.5–1 hour; *χ*^2^ = 5.0, *p* = 0.052), respectively, while the median CPT for *Ae. aegypti* was, as for PMD, a maximum of 0.5 hour. In summary, the CPTs very much reflect the observations made from the landing rates (Figs 2 and 3).

The observation that both repellent formulations showed a much longer complete protection under field conditions may be explained by the fact that landing rates measured in the field were magnitudes lower than in the AIC test (Fig. 3).

### Relative protection over time

**Figure 5:**
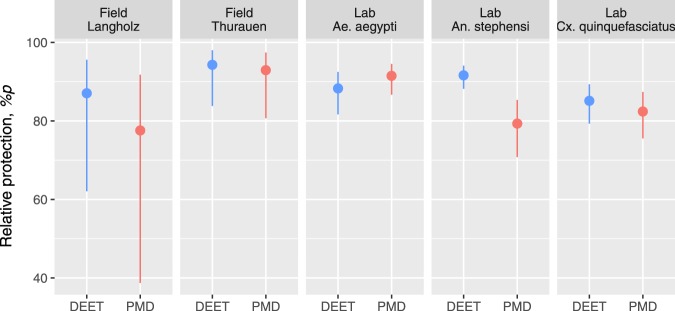
Relative protection *(%p)* over 6 hours for 15% PMD and 15% DEET. *%p* was calculated using Equation (1) on the bases of landing rates estimated by individual GLMs for each experiment. The points show the average *%p*, while the lines indicate the 95% CIs of the point estimates.

In agreement with CPT, *%p* over the 6 hours period reveals a similar picture in that the relative protection was similar between 15% PMD and 15% DEET with the only exception of the laboratory experiment in *An. stephensi* where PMD provided less protection than DEET (RR_DEET-PMD_ = 1.15; *p* < 0.001; Fig. 5). Intriguingly, when comparing the relative protection between the laboratory and field experiments, average protection was fairly similar although the results from the field showed somewhat higher variability, particularly in the experiments in the Thurauen Nature Reserve, reflected by larger 95% CIs.

## Discussion

The results from the comparative study between the AIC test and the HLC method in the field revealed comparable efficacy for 15% PMD and 15% DEET with the exception of DEET being superior to PMD against *An. stephensi* in the AIC test. The median CPTs in the AIC tests ranged between 0.5 and 1 hours for 15% PMD and between 0.5 and 2 hour for 15% DEET, while the CPTs measured in the field were at least 6 hours for both 15% DEET and 15% PMD. In contrast to the CPTs, *%p* did not vary greatly between the laboratory and field experiments. Intriguingly, irrespective of whether the repellent efficacy is estimated by CPT or *%p* the relative outcome between 15% PMD and 15% DEET remains the same.

Several previous studies have compared the duration of protection provided by PMD compared to DEET in biting mosquitoes using human subjects, both in the laboratory and in the field, and found that PMD shows comparable efficacy to DEET^13–16,18,27^. A caveat of those studies is, however, that the formulations were all commercially available products, also containing additional compounds that may influence the efficacy outcome in one or the other way. It is also noteworthy that in most studies the amount contained in the formulations differed between the PMD and DEET products, making a side-by-side comparison even more complex. The present study took these weaknesses of the earlier studies into consideration and compared two formulations that differed only in the active ingredient itself. That is both formulations evaluated consisted of ethanolic solutions at the same concentration of either PMD or DEET; hence the present study provides important information to the debate of alternative repellents to DEET and confirms that PMD provides a high level of protection that is almost as good as for DEET.

In the present study, the CPTs were clearly associated with the intensity of landing rates. In the laboratory, where the landing rates were magnitudes higher than in the field, the CPTs were also much shorter. A similar relationship is also seen when inspecting the landing rates across the AIC tests in that the shortest CPTs against DEET as well as against PMD were observed in *Ae. aegypti*, the mosquito species also showing the highest landing rates. A similar observation has previously been made by Barnard *et al*.^39^ who found for *Ae. aegypti* and *An. quadriannulatus* that the mean duration of protection from mosquito bites is linked to the initial biting rates in the controls.

The relationship between landing pressure and CPT is of some concern in the present study because the landing rates observed during the field tests were rather low as compared to previous field efficacy trials. For example, Carroll and Loye^14^ recorded from their field trial in California biting rates of 1.5 bites per minute on the untreated arm and 3 bites per minute on the untreated leg. In their field trial in northern Thailand, Champakaew *et al*.^40^ recorded a mean collecting rate of 2-204.2 mosquitoes per 20 minutes. While the WHO guidelines do not state the minimum landing pressure required to initiate or continue a repellent test, the US EPA guidelines are stricter on that subject and recommend a minimum of one mosquito landing within one minute. Granett^41^ even suggested a minimum biting rate requirement of one bite within 3 seconds on an exposed arm, irrespective of whether the experiments are carried out in the laboratory or in the field. According to the above references, the landing pressures measured in the current study would be too low. Unfortunately, the summer 2015 was exceptionally dry^42^, explaining to some extent the relatively low numbers of mosquitoes caught by the HLC. However, the landing rates observed here are on par with published data from southern England^43^ where average landing rates across different sites ranged between 0.00014 and 0.0028 landings per second, suggesting that such low landing rates might not be uncommon in the European context and simply represent the ecological reality of mosquito biology in Switzerland.

Mosquito landing rates may be influenced by various factors, including mosquito species^33^, diurnal activity patterns and mosquito density. Due to the relatively low numbers the data from the present study do not allow for measuring and comparing landing activity between species but the mosquito species most frequently caught were *Ae. vexans* and *Ae. cinereus/geminus*. *Ae. vexans*, a highly anthropophilic mosquito species^30^ was more prominent in the Thurauen Nature Reserve, while *Ae. cinereus/geminus* was predominant in the Langholz forest. This observation matches with the habitat preference and biting behaviour described for these species. *Ae. vexans* predominantly breeds in high numbers in temporarily inundated areas such as meadows as are present in the Thurauen Nature Reserve and is known to have a large flight range up to several kilometres^33^. In contrast, the sibling species *Ae. cinereus and Ae. geminus* prefer the edges of semi-permanent, partly shaded pools^33^. *Ae. vexans* is an important nuisance mosquito and could potentially act as a bridge vector for West-Nile virus^30^ as it also bites birds and has been shown to be a competent vector under laboratory conditions^44,45^. The vector potential of *Ae. cinereus/gemininus* is less known.

In addition to the landing rates, efficacy of a repellent also depends on the sensitivity of the test species. For example, Roey *et al*.^46^ found that the efficacy of picaridin repellents differed among Southeast Asian vectors of malaria and arboviruses. Importantly, a key difference between the present field and laboratory tests was the presence of mosquito test species. While the mosquitoes in the field are indigenous to Switzerland, the test species in the laboratory AIC were three highly anthropophilic mosquito species from the tropics that are recommended by WHO for testing topical repellents^20^. *Ae. aegypti* is a very aggressive, anthropophilic mosquito species that shows low sensitivity to repellents^47^, while *Cx. quinquefasciatus* is generally less active in the AIC test.

As field tests are time and cost intensive there is a lot of debate as to what extent field studies might be replaced by AIC test. In general, biting rates in the AIC test were extremely high compared to the biting rates encountered in the HLC, suggesting that the laboratory assays rather simulate extreme conditions. Therefore, the AIC test may be seen as a very conservative test. Most likely, repellents showing protection under the harsh AIC setting might actually reduce biting even better under field conditions, at least as long as the formulation is not mechanically rubbed or washed off. At first glance the results would suggest that the AIC might only have to be calibrated against the landing rates observed in the field. However, as mosquitoes show differential sensitivity to repellents this might be a rather naïve assumption. Therefore, it would be desirable to study the relationship between landing rates and repellent efficacy in more depth before modifying the AIC test in an attempt to adapt it more closely to the situation in the field.

## Conclusions

DEET slightly outperforms PMD under laboratory conditions, while PMD and DEET show similar efficacy over 6 hours against outdoor biting mosquitoes in Switzerland, irrespective of the ecological setting. Both CPT and %*p* over time provide a similar picture with reference to comparative protection efficacy between PMD and DEET, while CPT is linked to the observed landing rates. As the AIC set-up with the more anthropophilic mosquito species and high numbers yields very high landing rates, the AIC test in its current form may underestimate the actual efficacy of a topical mosquito repellent in areas with low to moderate mosquito landing pressure.

## Electronic supplementary material

Supplementary Material

## Data Availability

The datasets generated during and/or analysed during the current study are available from the corresponding author on reasonable request.
