# Supplementary material for: Evaluation of standard field and laboratory methods to compare protection times of the topical repellents PMD and DEET
Source: Sci Rep. 2018 Aug 22;8:12578. doi: 10.1038/s41598-018-30998-2 (PMC6105713; doi:10.1038/s41598-018-30998-2)
Supplement: Supplementary file 1 — Supplementary Material [file 41598_2018_30998_MOESM1_ESM.pdf]

Supplementary Material

Evaluation of standard field and laboratory methods to compare protection times of the topical repellents PMD and DEET

Barbara Colucci and Pie Müller

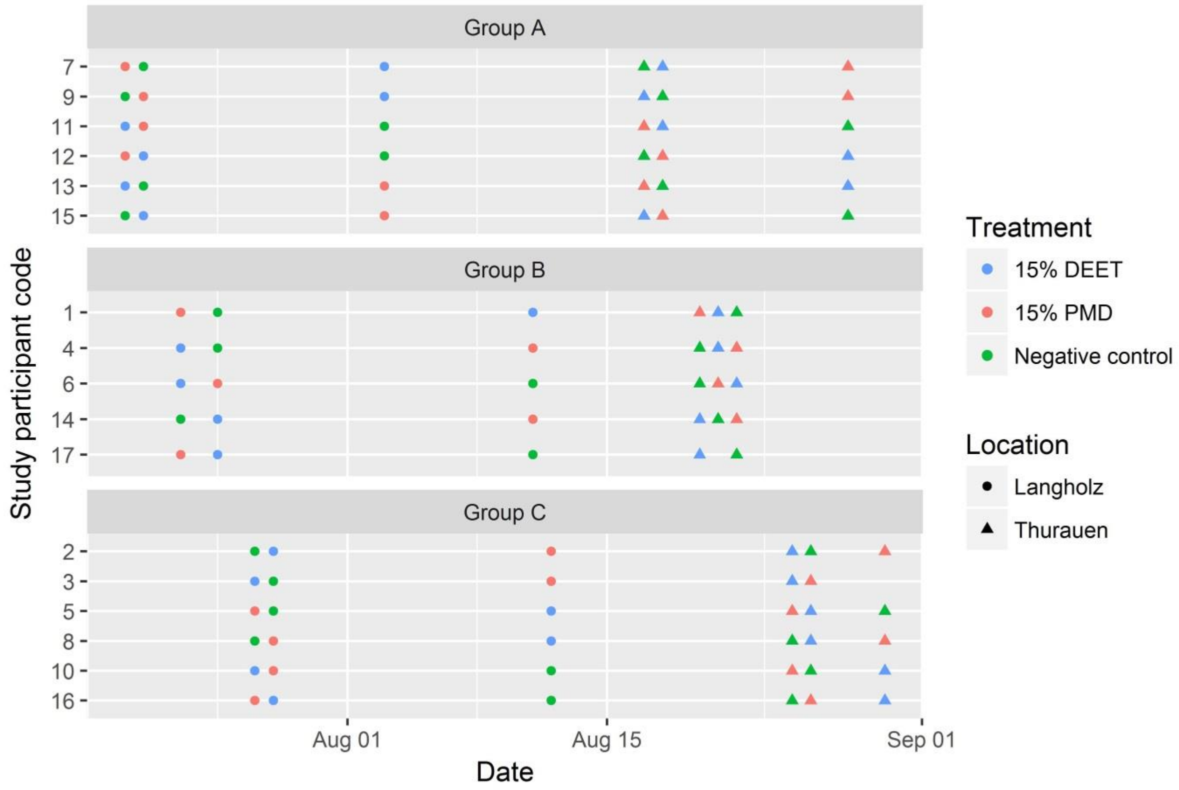

Supplementary Figure S1. Rotation scheme for the treatments in the field experiments. Each study participant was received each treatment (i.e. 15% DEET, 15% PMD and the negative control) once on three different days in each field site. The 18 participants were split into 3 groups, A, B and C. However, from the 18 participants enrolled in the study, one person dropped out, therefore, only 5 participants were included in group B.

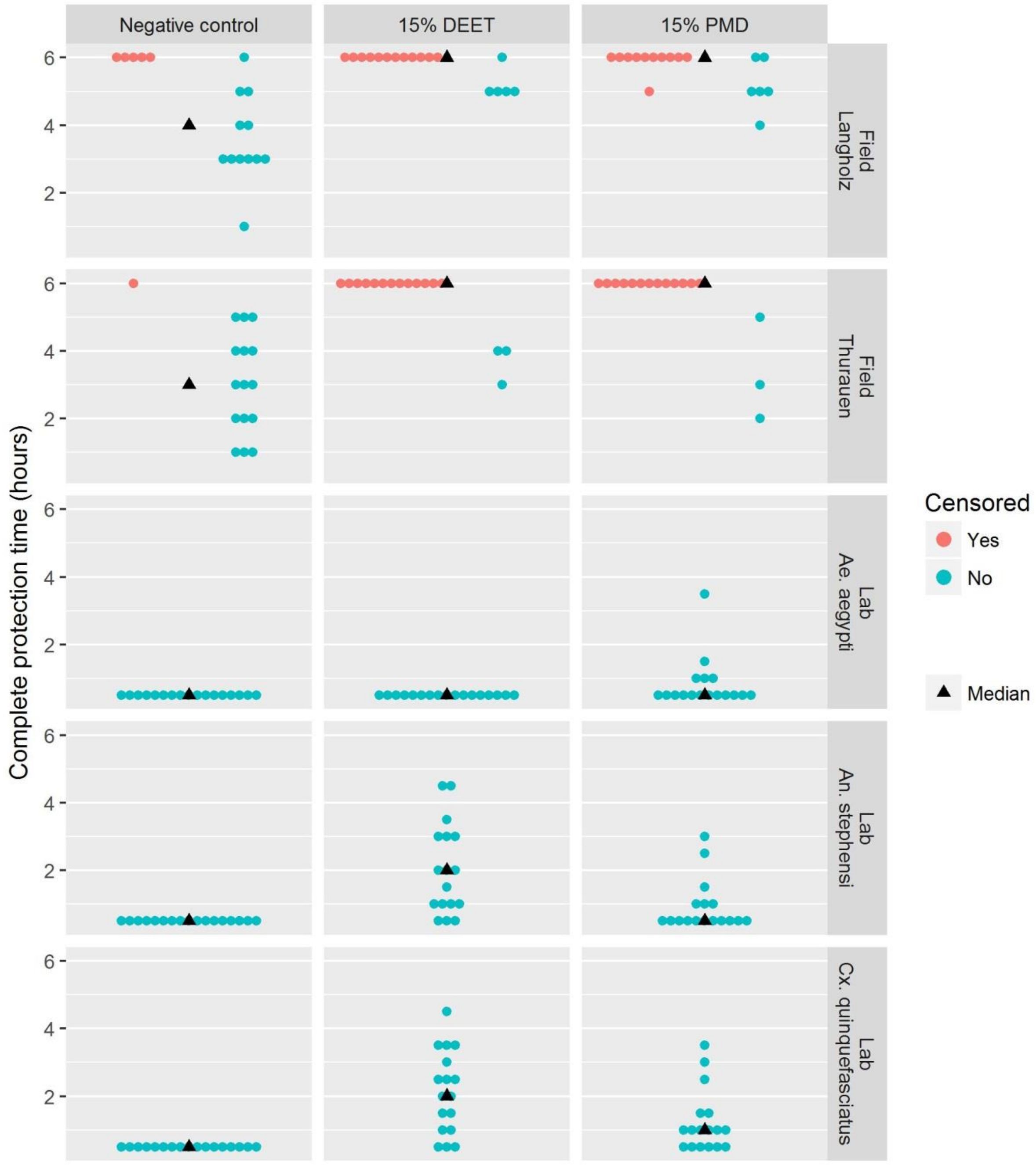

Supplementary Figure S2. Complete protection time (CPT). The CPT is the time from the application of the repellent till the first landing of a mosquito on the treated area. „Censored" means that there was no landing observed before concluding the experiment at 6 hours post application. The points show the measured CPTs for each experiment and study participant.
